# News and Coming Events

**Published:** 1909-01-02

**Authors:** 


### News and Coming Events.

Dr. Edward Emrys-Roberts, M.B., Ch.B. Vict., M.B., Ch. B. Liverp., has been appointed Pathologist to the Bristol General Hospital.

There are stated to be three candidates for the Vice-Chancellorship of the National University of Ireland, Professor George Sigerson, Fellow of the Royal University of Ireland; Dr. Windle, President of Queen's College, Cork; and Sir Christopher Nixon. The appointment, according to the *Times*, will probably be made at the next meeting of the Senate in January.

The medals, prizes, and certificates awarded during the winter session 1907-8 and the summer session, 1908, to the students of the Charing Cross Hospital Medical School were presented on the afternoon of December 18 in the out-patients' hall of that institution by Mr. John H. Morgan, F.R.C.S., consulting surgeon to the hospital. Dr. F. W. Mott, F.R.S., chairman of the School Committee, presided.

The Therapeutical and Pharmacological Section of the Royal Society of Medicine will hold a meeting at 20 Hanover Square, W., on Tuesday, January 5, 1909, at 4.30 p.m., when Professor A. R. Cushing, F.R.S., will read a paper on "Tissue Antiseptics with Special Reference to Animal Infections"; Dr. Alexander Haig on "Salicylates as Retentives"; and Dr. H. H. Dale on "Note on Nutmeg Poisoning."

The King has been pleased to appoint Dr. Harold Robert Dacre Spitta, M.D., B.S., D.P.H. Cambridge, to be Bacteriologist to his Majesty's Household. The post is a new one. Dr. Spitta is assistant lecturer on bacteriology and lecturer on clinical pathology at St. George's Hospital. He has also held the positions of lecturer on public health and hygiene, superintendent of the Clinical Investigation Department at St. George's Hospital, and has been bacteriologist to the Metropolitan Police, the Grosvenor Hospital for Women and Children, St. Mark's Hospital, City Road, and the General Lying-in Hospital, York Road.

Mr. William Spencer Lightfoot, R.N., M.R.C.S. Eng., L.S.A., who died on December 14, qualified in 1876, and joined the Royal Navy as a surgeon in 1880. He was on board the *Decoy* at the bombardment of Alexandria, July 11, 1882, and for his services during the Egyptian War he received the medal with clasp for Alexandria and the Khedive's bronze star. In the same vessel, in 1884, he was present during the naval operations near Suakin, in the Eastern Sudan, and received the Suakin clasp. In 1896 he was promoted to fleet surgeon, and in 1901 to Deputy-Inspector of Hospitals and Fleets. He retired from active service in 1904.

King Edward's Hospital Fund, the Metropolitan Hospital Sunday Fund, and the Hospital Saturday Fund have issued a fresh circular upon the revised uniform system of accounts. The circular, which has been drafted with the advice and approval of Mr. John G. Griffiths, takes the form of Supplementary Notes dealing with questions which have arisen out of the first year's experience of the revised system, and includes amended forms of income and expenditure account, balance-sheet, and statistical tables of expenditure upon in-patients and out-patients. The amended regulations are binding upon institutions applying for grants from either of the Funds, and the circular will be indispensable to all hospitals which have adopted the revised uniform system.

The Treasurer of St. Bartholomew's Hospital has received from the Worshipful Company of Salters £210, being the first instalment of a grant of 1,000 guineas voted to the hospital.

Surgeon-General John Edward Tuson, M.D., F.R.C.S., died recently at Eastbourne in his 79th year. After studying at St. George's and Middlesex Hospitals he qualified in 1851 and joined the Bengal Medical Service in 1853. He took part in the campaign on the North-West Frontier of India in 1855, obtaining mention in despatches, and during the Indian Mutiny he was engaged at the disarming of the Mooltan population, in the operations in Rohilcund, and in the action of Nugeena, being mentioned in despatches, and receiving for his services the thanks of the Commander-in-Chief and the Mutiny medal. He was also again mentioned in despatches and received the medal with clasp for his participation in the Mahsud Waziri Expedition of 1860. He retired from the Indian Medical Service with the rank of surgeon-general in 1884.

We learn that the London Mineral Water Trade Protection Association, a body which includes mineral water manufacturers of London and the country around, has taken official action in reference to the recent statements as to the purity of mineral waters. Acting in conjunction with Dr. Collingridge, the chief medical officer of health of the City of London, the association has issued a certificate to its members embodying the requirements of Dr. Collingridge for securing the absolute bacteriological purity of aerated waters, including soda water. That certificate, issued with the approval of Dr. Collingridge, ensures that the beverages have been tested and approved by the accepted bacteriological experts, and that the sanitary condition of the factories has been found satisfactory by the medical officer of health. Already certificates have been granted to forty-two firms.

The Councils of the Irish Medical Schools' and Graduates' and the Scottish Medical Diplomates' Associations inform us that a conjoined meeting of the two Associations will be held on Thursday, January 21, 1909, at 4 o'clock at the Hotel Cecil, to consider the exclusion of graduates of the Universities and the diplomates of the Scottish and Irish Corporations from candidature for positions on the staffs of certain hospitals in England. The following resolutions will be proposed at the meeting: (1) "That the present exclusion of all persons who do not hold certain qualifications from candidature for honorary positions on the staff of a public hospital is contrary to the public weal, and is a restriction which is not to the interest of the institution itself, excluding as it does all other candidates who may have exceptional claims to fill such positions." (2) "That the advertisements in the columns of the lay Press that honorary offices in certain hospitals are open only to those holding the diplomas of some particular corporation gives to the public the impression that such restrictive regulation is justified by the exceptional character of such qualification, which is not in accordance with fact." (3) "That these resolutions be forwarded to such persons as the Councils of the two Associations may determine." It is proposed to hold a banquet at the Hôtel Cecil on the night of the meeting. Tickets, 7s. 6d. each, can be obtained from either of the Hon. Secretaries, Mr. T. Hobbs Crampton or Mr. P. H. Parsons, on application to them at 11 Chandos Street, Cavendish Square, W.

